# Piperine Attenuates TBI-Induced Seizures via Inhibiting Cytokine-Activated Reactive Astrogliosis

**DOI:** 10.3389/fneur.2020.00431

**Published:** 2020-06-04

**Authors:** Yabei Song, Caiyun Cao, Qiuyue Xu, Simeng Gu, Fushun Wang, Xi Huang, Shijun Xu, Erxi Wu, Jason H. Huang

**Affiliations:** ^1^Department of Pharmacy, Nanjing University of Chinese Medicine, Nanjing, China; ^2^Department of Psychology, School of Medicine, Jiangsu University, Zhenjiang, China; ^3^Institute of Brain and Psychological Sciences, Sichuan Normal University, Chengdu, China; ^4^School of Pharmacy, Chengdu University of Traditional Chinese Medicine, Chengdu, China; ^5^Department of Neurosurgery, Baylor Scott & White Health, Temple, TX, United States; ^6^Department of Surgery, College of Medicine, Texas A&M University, Temple, TX, United States

**Keywords:** piperine, traumatic brain injury, seizure, glial fibrillary acidic protein, astrocytes, glymphatic system

## Abstract

Peppers have been used in clinics for a long time and its major component, piperine (PPR), has been proven to be effective in the treatment of seizures. The purpose of this study was to investigate the effects of piperine on early seizures in mice after a traumatic brain injury (TBI) and to explore the mechanism of the drug against the development on TBI. Specific-pathogen-free-grade mice were randomly divided into six dietary groups for a week: control group, TBI group, three piperine groups (low PPR group with 10 mg/kg PPR, medium PPR group with 20 mg/kg PPR, and high PPR group with 40 mg/kg PPR), and a positive control group (200 mg/kg valproate). Except for the control group, all the other groups used Feeney free weight falling method to establish the TBI of closed brain injury in mice, and the corresponding drugs were continuously injected intraperitoneally for 7 days after the brain injury. The results from behavior and electroencephalogram showed that piperine attenuated the subthreshold dose of pentylenetetrazole-induced seizures compared with the TBI group. The western blot results showed that the expression levels of inflammatory factors tumor necrosis factor-α (TNF-α) and interleukin-1β (IL-1β) were reduced by piperine. The immunostaining results showed that the brain-derived neurotrophic factor (BDNF) was also reduced by piperine. In addition, positive cell counts of astrocytic fibrillary acidic protein (GFAP) in immuno-fluorescence showed that they were also reduced. Our data show that piperine treatment can reduce the degree of cerebral edema, down-regulate TNF-α, IL-1β, and BDNF, decrease the reactivity of GFAP in the hippocampus, and inhibit TBI-induced seizures.

## Introduction

Traumatic brain injury (TBI) is a major cause of death and disability worldwide. It triggers many neurological complications, such as epilepsy, depression, and dementia (1). Seizure is a common and debilitating phenomenon of traumatic brain injury (2), and it is usually caused by cytotoxic and vasogenic edema, oxidative stress, and inflammation (3). Neuroinflammation is a major pathological process in the TBI, and it is due to the increased permeability of the blood–brain barrier (BBB) to allow the passage of big molecular and blood cells. These intruders and neural cellular debris initiate an inflammatory cascade in local microglia and astrocytes to release inflammatory cytokines, chemokines, and growth factors (4). Many proteins are activated in order to compensate the injury, such as NKCC1, AQP4, glial fibrillary acidic protein (GFAP), etc., (5, 6). The astrocytes are activated to extend their processes to form a barrier around the lesion area, which consists majorly of astrocytes, endothelial cells, and extracellular matrix. This barrier is the primary form of glia scar (7), which is commonly seen after TBI, and a major reason for seizures. Many cytokines are involved in the process, such as transforming growth factor β (TGF-β), interleukin-1β (IL-1β), tumor necrosis factor-α (TNF-α), fibrinogen, etc., (8). These astrocytic barriers act as a physical barrier to encapsulate damaged tissue in order to prevent toxic molecules from the injured tissue; however, reactive astrogliosis has been proven as the major reason for seizures (5). Early seizure happens in nearly half of TBI incidences, depending on the severity of the TBI, and seizure in turn makes TBI even worse because of energy stress. Thus, it is critically important to treat TBI-induced seizures.

Despite that there are more than 20 different anti-seizure drugs, approximately a third of epilepsy patients remain resistant to pharmacotherapy (9). In addition, the currently available drugs in clinical use are not specifically for early seizures induced by TBI; safer and more effective drugs to treat early seizures induced by TBI are needed urgently. Traditional Chinese medicine has a long history of treating epilepsy, with stable curative effect and small side effects. More than 1,400 years ago, “Compendium of Materia Medica” recorded that pepper was effective in the treatment of epilepsy. Black pepper (*Piper nigrum*) is widely used as a functional food and has a broad range of applications, such as food additive, cosmetic, and traditional medicine. Even though black pepper has a long history in clinical use for treating epilepsy, its mechanism is still not clear. Piperine (PPR), a piperidine alkaloid, is a major component of numerous pepper plant families (10), especially in peppers (11). It can be used to treat abdominal pain, vomiting, and diarrhea, and loss of appetite. Modern pharmacological studies have shown that piperine has a variety of effects, including anti-oxidant (12), immune regulation (13), anti-tumor (14), drug metabolism-promoting effects (15), and mood and cognitive disorders (16). Piperine has a large safety range, and its chemical structure is different from common anti-epileptic drugs. Piperine can exert effects in many brain diseases because of its efficient brain uptake and high membrane permeability (17); for example, it was found that piperine can block NaV channels or TRPV1 instead of Ca^2+^ channel, and it is expected to become a new type of broad-spectrum anti-epileptic drug (18). In addition, piperine was also shown to significantly block convulsions induced by the intracerebroventricular injection of threshold doses of kainite, but with no or only slight effects on convulsions as induced by L-glutamate, N-methyl-D-aspartate, or guanidinosuccinate (19). However, it is not clear if piperine is effective in TBI-induced seizures. The aim of the present study was to evaluate the anti-convulsant effects of piperine in mice with TBI-induced epilepsy. In addition, we also examined the expression of inflammatory factors and the expression of GFAP in order to explore the mechanism of its anti-epileptic effects.

## Materials and Methods

### Drugs and Chemicals

Piperine and Tween-80 were purchased from Nantong Feiyu Biotechnology Co., Ltd., and the piperine batch number was FY1327B0629. Piperine is difficult to dissolve in water. When using it, 2% Tween-80 solution is used to prepare suspensions with different concentrations. The antibodies for TNF-α (cat. no. H052), IL-1β (cat. no. H002), GFAP (cat. no. H076), and brain-derived neurotrophic factor (BDNF) (cat. no. E11-0774B) were from Enjing Biotechnology.

### Animals and Experimental Procedures

A total of 60 specific-pathogen-free male Institute of Cancer Research mice (*n* = 60) were obtained from the Qinglongshan Animal Breeding Farm, Jiangning District, Nanjing, Co., Ltd. [Animal License No. SCXK (su) 2017-0001], China, and raised in the experimental animal center of Nanjing University of Chinese Medicine. During the experiment, the mice were housed in plastic cages in a controlled environment (23 ± 1°C; humidity, 55 ± 5%) under a 12-h/12-h light/dark cycle, and food and water were supplied freely. The animals were treated in accordance with the guidelines set by the National Institutes of Health (Bethesda, MD, USA) for the humane treatment of animals, and this study was approved by the Experimental Animal Ethics Committee of Nanjing University of Traditional Chinese Medicine.

The animals were randomly divided into six groups: (i) control group (*n* = 10), the mice in this group were administered with 0.9% normal saline; (ii) epilepsy group (*n* = 10), after TBI-induced epilepsy, the mice in this group were administered 0.9% saline; (iii) piperine group (*n* = 30), which was divided into three sub-groups, the mice in these groups were orally administered with 10, 20, and 40 mg/kg piperine for 7 days; the dose used was according to previous reports (18); and (iv) positive group, the mice in this group were administered with 200 mg/kg valproate.

### Induction of Epilepsy in the Mice After TBI

The TBI was prepared by methods referring to the weight falling method, as reported before in our previous paper (5). Briefly, the mice were fixed on a stereoscopic experimental table and maintained at a continuous anesthesia state by 2.5% inhaling isoflurane. The end of the striking instrument was aligned with the left parietal bone of the mouse. One 20-g steel ball was dropped freely from a 30-cm-high stainless steel tube.

**Figure:**
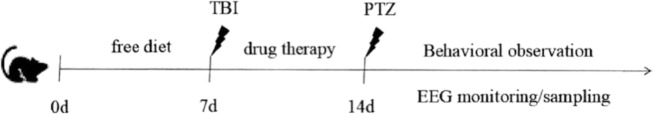


### Behavioral Test

The mice in each group were given an intraperitoneal injection of sub-convulsive dose (37.5 mg/kg) of pentylenetetrazol (PTZ) at 2 h after the last administration of drugs to detect their seizure susceptibility to epileptogenic agents. The mice were placed in a transparent organic plastic empty cage and observed for 30 min. The seizure latency of clonic seizure (equivalent to grades 1–3), generalized tonic seizure latency (up to grades 4–5), number of seizures, average seizure intensity, and other behavioral parameters were recorded. The seizure intensity of epilepsy was graded according to the Racine criteria as grade 0—no response, grade 1—rhythmic mouth, ears, or facial muscle twitching, grade 2—twitching of neck muscles, frequent nodding, and tail erection, grade 3—spasticity of one forelimb but not accompanied by rearing, grade 4—bilateral forelimb spasticity, with hindlimb straightening and body erection, and grade 5—generalized tonic–clonic seizures with loss of postural control or death from twitching. Among them, the mice with persistent severe seizures can be terminated by an intraperitoneal injection of diazepam at 10 mg/kg.

### EEG Test

Five mice in each group were anesthetized for the brain surgery. First, the skin and the subcutaneous tissue on the skull surface were removed, and after the skull was exposed, a window was opened on the posterior side of the bregma and on the right side of the raphe of the mouse with a hand-held cranial drill. The size of the bone window was about 3 × 3 mm to expose the brain tissue. A steel electrode was inserted into the exposed cortical tissue. After the animal recovered, electroencephalogram (EEG) monitoring with Pclamp10.4 data analysis software was performed for 1 h every day.

### Brain Tissue Water Content Test

The drug was administered for 7 days, and the TBI was established according to the abovementioned method, except for the control group, after the last administration. At 24 h after TBI, the mice were sacrificed by cervical dislocation and the brain tissues were rapidly harvested. The brains were placed on tin foil paper to weigh the brain wet weight and then placed in an oven at 80°C for baking for 48 h. The brain wet and dry weight method was used to calculate: water content of brain tissue = (wet weight–dry weight)/wet weight ×100%.

### Immunohistochemistry Assay for GFAP and BDNF in the Hippocampus

After the last EEG recordings, the mice were fixed in supine position on a dissecting table, and after the mouse blink reflex disappeared with isoflurane, the animals were sacrificed. The brain tissues were fixed in 4% paraformaldehyde for 24 h at 4°C. The brains were removed after dehydration, embedded in optimal cutting temperature compound, and processed for coronal serial cryosectioning with a slice thickness of 40 μm. Serial sections of mouse brain tissues were cut at a thickness of 4 μm. The sections were sequentially washed in xylene I15 min-xylene II15 min-absolute ethanol I5 min-absolute ethanol II5 min-85% alcohol 5 min-75% alcohol 5 min-distilled water. Tissue sections were placed in retrieval cassettes filled with ethylenediaminetetraacetic acid target retrieval buffer (pH 8.0) for antigen retrieval in a microwave oven. After natural cooling, the slides were placed in phosphate-buffered saline (PBS) (pH 7.4) and washed by shaking three times for 5 min on a decolorizing shaker. The slices were slightly spin-dried and circled around the tissue using a histochemical pen (to prevent the antibody from flowing away), an autofluorescence quencher was added to the circle for 5 min, and the circle was rinsed with running water for 10 min. BSA was added dropwise in the circle and incubated for 30 min for serum blocking, with the blocking solution gently shaken off and the GFAP and BDNF antibody drips were prepared in a certain proportion with PBS on the sections. The sections were placed flat in a wet box for overnight incubation at 4°C. On the next day, the sections were washed in PBS and slightly dried. The tissues were covered dropwise with secondary antibodies with the corresponding species of the primary antibody in a circle and incubated in the dark for 50 min.

### Western Blot Analysis

Following the homogenization of the brain tissues and the protein extraction, the protein concentration was measured with a bicinchoninic acid assay protein concentration assay kit after homogenization of the hippocampal tissue, the proteins were separated by electrophoresis on 10% polyacrylamide gel (sodium dodecyl sulfate-polyacrylamide gel electrophoresis), and then the proteins were transferred to a polyvinylidene fluoride membrane and then blocked with 5% skim milk in Tris-buffered saline containing Tween-20 (TBST) for 1 h. The membranes were incubated overnight at 4°C with anti-tumor necrosis factor-α (TNF-α, dilution, 1:1,000), anti-Interleukin-1 beta (IL-1β, dilution, 1:1,000), and anti-BDNF (IL-1β, dilution, 1:1,000). Subsequently, the membrane was incubated with goat anti-mouse immunoglobulin G secondary enzyme. After 1 h of incubation and washing with PBS, electrochemiluminescence solution was added and exposed to a Tencent gel imaging system for developing and photographing, and the optical density values of the target bands were analyzed using an Alpha software.

### Statistical Analysis

The measurement data were expressed as mean ± standard deviation (x ± s), and statistical analysis was performed using SPSS 17.0. Paired *t*-tests were used for within-group comparisons, and one-way analysis of variance was used for between-groups comparisons, with *p* < 0.05 considered as statistically significant.

## Results

### Effects of Piperine on Seizure Latency in Mice After TBI

The mice in the control group showed no obvious abnormalities, and some of them had convulsive behavior after a subthreshold dose of PTZ treatment, but they were all below grade 2. The animals in the TBI group showed an incubation latency of about 78.8 ± 6.4 s (*n* = 10). The behaviors were manifested as forearm tremor, hind limbs standing upright, and falls. Extensive generalized tonic seizures and several major seizures occurred within 30 min; most animals in the other groups of mice had recurrent episodes of grades 2–4. Compared with the control group, the latency of tonicity and clonus in the TBI group was significantly shortened (*p* < 0.05, one-way ANOVA, *n* = 10), and the convulsion rate of mice was the highest. Compared with the TBI group, the number of seizures in the low-, medium-, and high-dose piperine groups was significantly reduced, and the seizure latency was significantly prolonged (*p* < 0.05, one-way ANOVA, *n* = 10, Table 1, Figure 1). The effect of high dose piperine was slightly weaker than that of the sodium valproate group.

**Table 1:** Effect of piperine on seizure latency in mice after a traumatic brain injury.

**Group**	**Dose/mg kg^**−1**^**	**Incidence of tonic–clonic seizures (%)**	**Clonic seizure latency(s)**	**Tonic seizure latency(s)**	**Mean seizure grade**	**Duration of convulsion(s)**
Con		20	130.2 ± 8.58	195 ± 11.31	2	20.65 ± 6.28
TBI		100	78 ± 6.16^##^	101.57 ± 9.22^##^	5	178.23 ± 7.26
Low PPR	10	78	86.7 ± 9.59	104.55 ± 12.58	4.5	142.48 ± 6.59
Medium PPR	20	45	105.6 ± 9.12^**^	120.56 ± 8.57^**^	3.6	11.75 ± 7.45
High PPR	40	60	113.8 ± 7.26^**^	160.2 ± 10.06^**^	4	77.45 ± 8.24
VPA	200	40	118.25 ± 6.95^**^	163.5 ± 9.19^**^	3.2	65.86 ± 7.43

** *p < 0.01 compared with TBI*;

## *p < 0.01, compared with control*.

**Figure 1:**
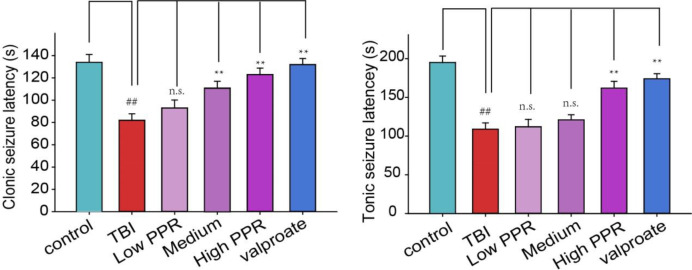
Effects of piperine on seizure latency in mice after a brain injury. ^**^p < 0.01 vs. the TBI group; ^##^*p* < 0.01 vs. the control group.

### The Effects of Piperine on the EEG of Mice After TBI

**Figure 2:**
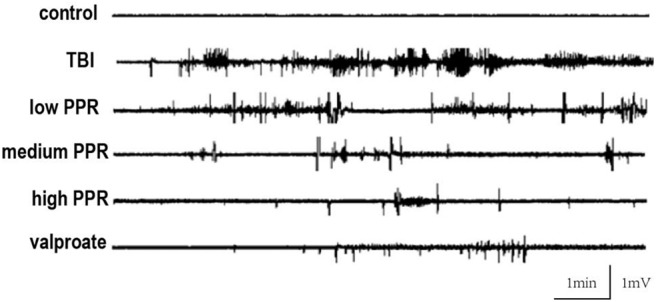
Effect of piperine on electroencephalogram (EEG) in epilepsy after a brain injury; typical recordings of EEG from the five groups.

Five mice in each group were randomly selected for EEG monitoring, after the TBI, for a period of 30 min every day, and 10 min EEG at seizure was intercepted for comparison. In the control group, the brain EEG waves of mice were mainly basal waves with a stable amplitude, and no obvious rhythmicity was observed, while the EEG of mice in the TBI group showed obvious paroxysmal epileptiform waves with an average discharge of more than four times per second, manifested as spikes, sharp waves, and slow waves with high potentials; the low-, medium-, and high-piperine-dose groups have different levels of spikes, and epileptiform spikes occurred in the high-dose group after a long latency period. The frequency of spikes in the three groups was not as high as that in the TBI group (Figure 2). Epileptic waves occurred occasionally in mice treated with sodium valproate, with amplitudes within 1 mV.

**Figure 3:**
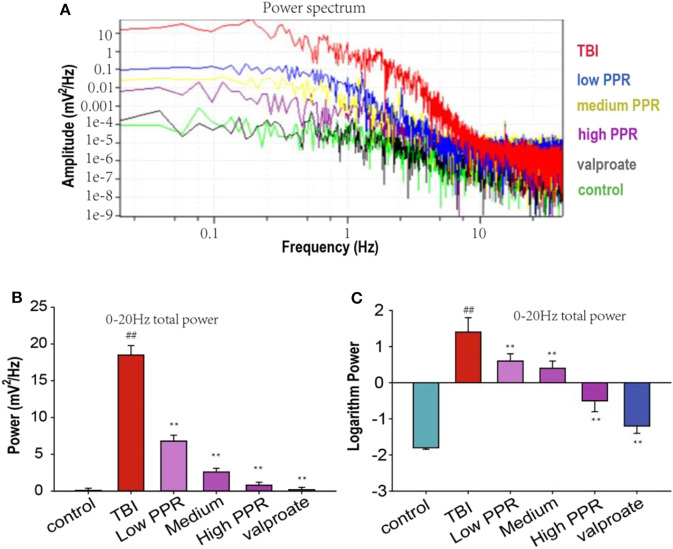
Effect of piperine on the total brain discharge in mice with epilepsy after a traumatic brain injury (TBI). ^**^p < 0.01 vs. the TBI group; ^##^*p* < 0.01 vs. the control group. **(A)** The upper panel shows the power spectrum of electroencephalogram (EEG) in the five groups. The lower panels **(B,C)** shows the analysis of the powers of EEG in the five groups (^**^p < 0.01).

The EEG in the same period of 60 s with obvious features was analyzed with a power spectrum using Clampfit 10.4 software for data analysis. The EEG discharges of the six groups, in descending order, were: TBI group > piperine low dose group > medium dose group > high dose group > sodium valproate group > control group. Through the statistics of the sum of brain discharges from 0 to 20 Hz, the results showed that the discharge of the TBI group was the largest, and the values of the other groups were smaller; for comparison, logarithmic transformation was used for data processing, and the values of the TBI group and the low-dose group were positive, and the values of the control group, high-dose group, and sodium valproate group were negative. In conclusion, a high dose of piperine could effectively inhibit the epileptiform discharges in mice, and there was statistical significance compared with the TBI group (*p* < 0.05, one-way ANOVA, Figure 3).

### The Effects of Piperine on Brain Water Content in Mice After TBI

After 24 h of TBI, compared with the control group, the water content of the brain tissue in all the groups increased in varying degrees after TBI (*p* < 0.01, one-way ANOVA, *n* = 10, Table 2). Compared with the TBI group, the animals in the groups treated with piperine showed reduced water content on brain tissue and degree of brain edema. Among them, the low-dose group has reduced brain water content, but there is no statistical significance; compared with the TBI group, the middle- and high-dose groups have significant differences (*p* < 0.05, Figure 4), which shows that piperine is conducive to the repair of the blood–brain barrier and can reduce the degree of brain edema.

**Table 2:** Effect of piperine on brain water content after brain injury.

**Group**	**Quantity**	**Brain wet weight (*g*)**	**Brain dry weight (*g*)**	**Difference in brain water content (%)**	**Brain water content (%)**
Control	10	0.3795 ± 0.0059	0.1135 ± 0.0014	0.2660 ± 0.0059	70.09 ± 0.57
TBI	10	0.0996 ± 0.0066	0.1003 ± 0.0026	0.2993 ± 0.0084	74.20 ± 0.98^##^
Low PPR	10	0.3892 ± 0.0091	0.1056 ± 0.0017	0.2836 ± 0.0104	72.84 ± 1.02^*^
Medicum PPR	10	0.3843 ± 0.0040	0.1067 ± 0.0024	0.2776 ± 0.0053	72.23 ± 0.77^**^
High PPR	10	0.3949 ± 0.0149	0.1110 ± 0.0032	0.2839 ± 0.0129	71.87 ± 0.76^**^
Valproate	10	0.3858 ± 0.0023	0.1105 ± 0.0017	0.2752 ± 0.0035	71.35 ± 0.54^**^

* *p < 0.05*,

** *p < 0.01 vs. the TBI group*;

## *p < 0.01 vs. the control group*.

**Figure 4:**
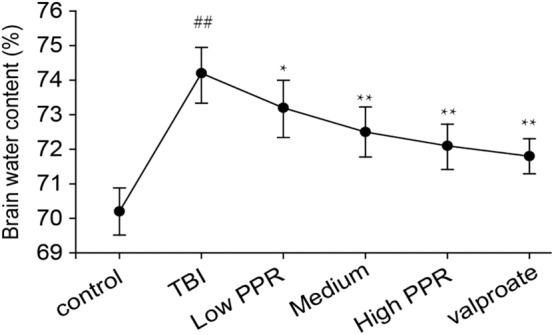
Effect of piperine on brain water content in epilepsy in mice after a brain injury. ^*^p < 0.05, ^**^p < 0.01; ^##^*p* < 0.01.

### Effects of Piperine on the Expression of Inflammatory Factors and Apoptotic Proteins in Mice With TBI

**Figure 5:**
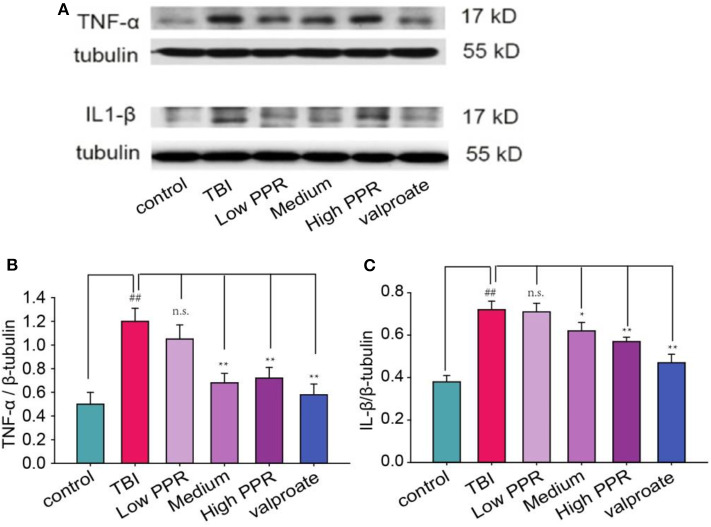
The anti-convulsant effects of piperine decrease the expression levels of TNF-α and IL-1β in mice with a traumatic brain injury. **(A)** The upper panel is a typical western blot. **(B,C)** The lower panels are the analysis for TNF-α and IL-1β, respectively. ^*^p < 0.05, ^**^p < 0.01 compared with the TBI group; ^##^*p* < 0.05, compared with the control group.

To probe into the mechanisms of piperine's therapeutic effects on TBI-induced seizure, we further tested the effects on the cytokines. Compared with the control group, the protein content of TNF-α and IL-1β in the TBI group was significantly increased (*p* < 0.01, one-way ANOVA, *n* =10, Figure 5); the low-, medium-, and high-dose groups had down-regulated expressions of TNF-α and IL-1β, especially in the high-dose group (*p* < 0.01). The treatment effect was slightly lower than that of the valproate group, and there was no statistical significance between the two groups (*p* > 0.05). Compared with the TBI group, the results of the low-dose group were not statistically significant (*p* > 0.05). The results showed that piperine inhibited TNF- α and IL-1β in a dose-dependent manner (*p* < 0.01, one-way ANOVA).

### Effect of Piperine on the Expression of BDNF in TBI Mice

**Figure 6:**
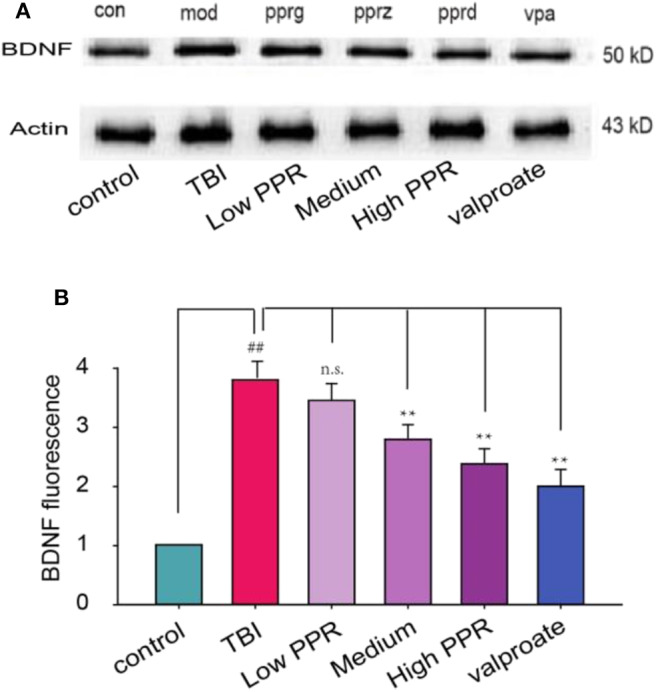
Effects of piperine on the expression of brain-derived neurotrophic factor (BDNF) in the hippocampus of mice with traumatic brain injury (TBI)-induced seizure. **(A)** The upper panel is a typical western blot for BDNF. **(B)** The lower panel is the statistical comparison among the groups. ^##^*p* < 0.01, compared with the control group; ^**^p < 0.01, compared with the TBI group; one-way ANOVA, *n* = 5.

In addition to the neuro-inflammation factors, neural growth factors have been suggested to increase. Here we tested the expression of BDNF in the hippocampus of TBI mice. The results from western blot showed that TBI induced the expression of BDNF, which was inhibited by piperine (Figure 6). Then, we used immunostaining to screen the distribution of BDNF and found that BDNF was not overlapped with GFAP (Figure S1). This suggested that the increased BDNF after TBI comes from many cell types, including astrocyte, microglia, or even neurons. Under high magnification, the experimental results showed that the fluorescence density of GFAP immunoreactivity increased significantly after TBI, but it is decreased to varying degrees with piperine. A statistical comparison showed that the TBI group showed the highest immuno- fluorescence density, and there was a significant difference compared with the control group (^**^p < 0.01, one-way ANOVA, Figure 6).

### Effect of Piperine on the Expression of GFAP in the Hippocampus of TBI Mice

**Figure 7:**
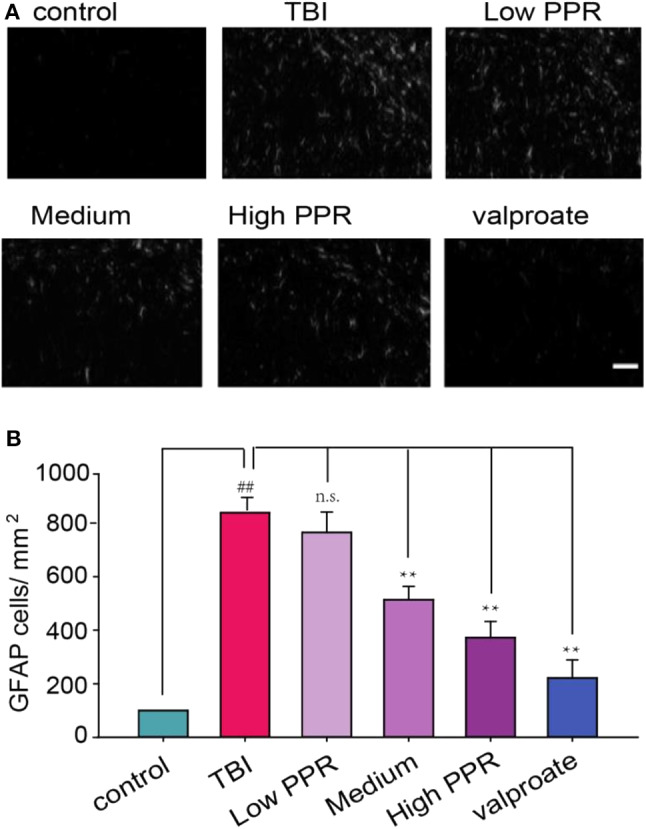
Effects of piperine on the expression of glial fibrillary acidic protein (GFAP) in the hippocampus of mice with traumatic brain injury-induced seizure. Scale bar = 100 μm. **(A)** The upper panel is a typical recording for the GFAP-positive cells in the hippocampus. **(B)** The lower panel is the statistical comparison among the groups (^**^p < 0.01, one-way ANOVA, *n* = 5); ^##^*p* < 0.05, compared with control.

These experiments suggest that piperine can attenuate TBI-induced seizures; we next probed into its neural target. Previous studies suggested that the TBI-induced release of cytokines can induce reactive astrocytes, which involves changes in GFAP (20). In addition, many other proteins are activated to compensate the injury, such as NKCC1 and AQP4 (5, 6). Under high magnification, the experimental results showed that the GFAP-positive cells in the hippocampal CA1 regions of the control group had smaller cell bodies, slender protrusions, and more branches. In the TBI group, due to the stimulation of brain injury, GFAP expression is very high as seen from the immunoreactivity staining. After a statistical comparison of positive cells, the TBI group showed the highest GFAP cells, and the difference was significant compared with the control group (*p* < 0.05, one-way ANOVA, Figure 7). In the PPR administration group, the high-dose group showed the smallest fluorescence density (*p* < 0.05) and showed a certain dose dependence. Although the low-dose group decreased, it was not statistically significant (*p* > 0.05).

## Discussion

TBI induces a variety of cellular biochemical processes, including blood–brain barrier destruction, brain edema, calcium overload, oxygen free radical overload, and release of a large number of inflammatory factors (21). Our previous studies found that TBI induced cytokine release, which can promote the expression of many proteins such as NKCC1 (5). NKCC1 is an important transporter to buffer intracellular Cl^−^. High NKCC1 can induce an overload of Cl^−^ intracellularly, and inhibitory GABAergic activity can be changed to excitatory to induce seizures (22). In addition, our previous study also showed that astrocytes play a significant role in the function of cell K^+^ (23). After the TBI, the astrocytes were damaged, the ability to recover K^+^ was weakened, the high-potassium/low-calcium state was formed outside the cells (24), the excitability threshold of the neurons decreased, and the excitability of the neurons was increased, leading to the occurrence of epilepsy (25, 26). In this study, we used a closed-head injury TBI, and the seizure was induced by a subthreshold dose of PTZ. We are the first to show the therapeutic effect of piperine on the susceptibility of mice to TBI-induced seizure according to behavior and EEG results. EEG is a very good way to monitor a seizure (27). The results showed that the treatment group could considerably prolong the latency of the seizure after brain injury, and the effect of the high-dose group was better than that of the low-dose group. Through the analysis of cortical EEG results, the EEG of the TBI group was found to be permanently changed in the blank group, and the spikes of the abnormal high frequency and the slow wave of the spine were displayed, which suggested that piperine can inhibit the TBI-induced seizures. The data also showed that the mechanism might be due to inhibiting TNF-α and releasing IL-1β, which induced reactive astrogliosis. Both microglia and astrocyte can be activated to release BDNF, TNF-α, and TGF-β after TBI (28). The increases of BDNF, TNF-α, and IL-1β might work together to enhance GFAP expression and induce astrogliosis. In this experiment, the number of GFAP-positive cells in the hippocampus of epileptic mice treated with traditional Chinese medicine was significantly decreased in a dose-dependent manner. GFAP is a specific cytoskeleton protein that maintains the basic morphology and the function of astrocytes and is highly sensitive to astrocyte damage (29). Therefore, the increased expression of GFAP, which can be used as a marker for judging the severity of brain injury, is the commonness of many epileptic TBIs (30).

Astrocytes are critical in maintaining physiological homeostasis in the brain and play an important role in keeping the BBB integrity by forming end feet around endothelial cells (31). The astrocytic buffering of ions and the absorption of glutamate decrease, and the induced accumulation of glutamate in extracellular spaces leads to seizures (32). In addition, reactive astrocytes are capable of producing cytokines, ATP, chemokines, and growth factors, which can in turn activate microglial responses. Microglial response was found to be dependent upon purinergic signaling. Microglia are specialized immune cells with phagocytic capabilities, which can actively phagocytose cellular debris. Microglia can also release IL-1β and TNF-α, which can induce long-term tissue degeneration after TBI (33). These cytokines in turn can induce astrogliosis, which involves the expression of GFAP, and extension of processes and swelling of cell bodies. A recent study conducted in a mouse CCI TBI reported hypertrophic astrocytes in the lesion area 3 days after TBI (34). Increased astrocyte activation influences the production of anti-inflammatory mediators and suppresses microglial activation. It is suggested that microglia can heighten their activity as a compensatory mechanism for the depleted astrocytic number, and the excessive activation of astrocyte can dampen microglial responses. It is found that astrocytes can affect the local environment after TBI by forming a physical barrier to encapsulate damaged tissue, but they have been proven to be the source of seizure.

Overall, reactive astrocytes are capable of producing pro-inflammatory cytokines, chemokines, and matrix metalloproteinase that degrade the extracellular matrix and cause further BBB disruption. Consistent with many previous reports which suggested that piperine has many pharmacological effects such as anti-inflammatory effects (35). Piperine is an anti-epileptic drug with a potential research value, and its anti-epileptic mechanism may be related to astrocyte regulation. However, many other specific molecular mechanisms remain to be further studied; for example, neuromodulators (norepinephrine and serotonin) have been suggested to be involved in the emotional functions of the brain (36) and have also been suggested to have anti-convulsant effects (37). What Mori et al. illustrated as the mechanism of anti-convulsant action, through increasing the 5-HT concentration in the cerebral cortex of mice by piperine, is really true (38). In addition, further studies are needed to study the effects of piperine on seizures and impaired cognitive and behavioral performance after TBI recovery. This might open up new ideas for better development of safe and effective anti-epileptic drugs. Consistently, it is suggested that even mild TBI can induce function network changes and cognitive and behavioral performance after TBI (33, 39).

## Data Availability Statement

All datasets generated for this study are included in the article/Supplementary Material.

## Ethics Statement

The animal study was reviewed and approved by Animal Ethics Committee of Nanjing University of Traditional Chinese Medicine.

## Author Contributions

YS, FW, SG, and JH planned the study. CC, QX, and SG did the investigation. EW, SX, and YS analyzed the data. YS, FW, SG, and XH wrote the paper.

## Conflict of Interest

The authors declare that the research was conducted in the absence of any commercial or financial relationships that could be construed as a potential conflict of interest.
