# Supplementary material for: Piperine Attenuates TBI-Induced Seizures via Inhibiting Cytokine-Activated Reactive Astrogliosis
Source: Front Neurol. 2020 Jun 4;11:431. doi: 10.3389/fneur.2020.00431 (PMC7325955; doi:10.3389/fneur.2020.00431)
Supplement: Supplementary file 1 [file Image_1.pdf]

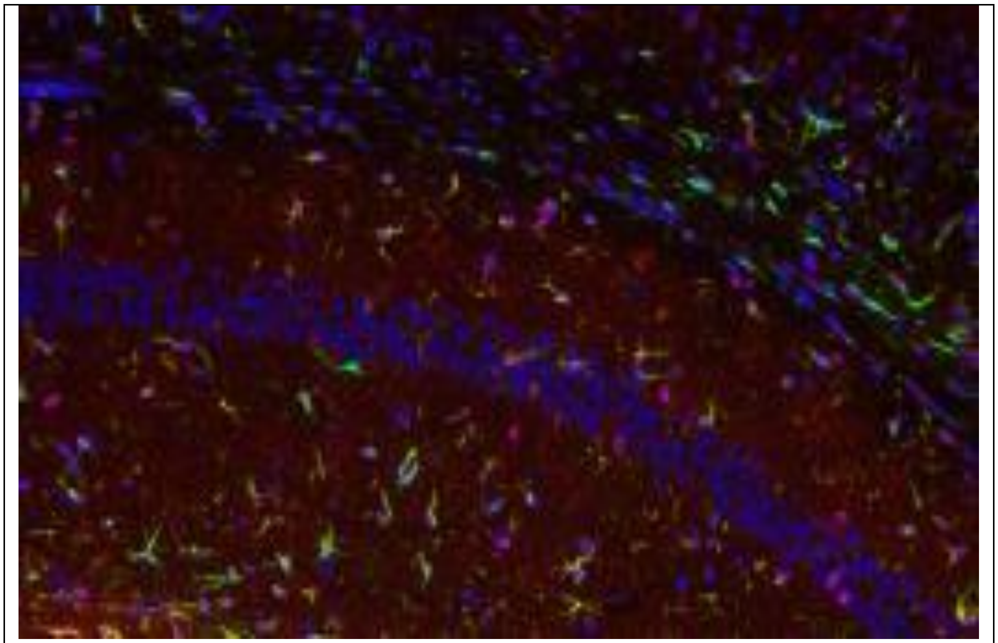

Fig S1. Immunostaining for GFAP (green), BDNF（red）, and Debi (blue) shows that BDNF cannot overlap with BDNF, which suggested that BDNF was released from many kinds of cells, including astrocyte and microglia.
